# Red cell transfusions as an independent risk for mortality in critically ill children

**DOI:** 10.1186/s40560-015-0122-3

**Published:** 2016-01-07

**Authors:** Surender Rajasekaran, Eric Kort, Richard Hackbarth, Alan T. Davis, Dominic Sanfilippo, Robert Fitzgerald, Sandra Zuiderveen, Akunne N. Ndika, Hilary Beauchamp, Anthony Olivero, Nabil Hassan

**Affiliations:** Department of Pediatric Critical Care Medicine, Helen DeVos Children’s Hospital, 100 Michigan St N.E., Grand Rapids, MI 49503 USA; Department of Pediatric Hospitalists, Helen DeVos Children’s Hospital, Grand Rapids, MI USA; Department of Pediatrics, Michigan State University College of Human Medicine. Grand Rapids, Grand Rapids, MI USA; Department of Research, Grand Rapids Medical Education Partners and Michigan State University, Grand Rapids, MI USA; Department of Surgery, Michigan State University, Grand Rapids, MI USA

**Keywords:** Pediatric critical care, Transfusion, Acuity scores, Mortality prediction, PRISM, PELOD, PIM2, Pediatric outcome scores

## Abstract

**Background:**

Severity of illness is an important consideration in making the decision to transfuse as it is the sicker patient that often needs a red cell transfusion. Red blood cell (RBC) transfusions could potentially have direct effects and interact with presenting illness by contributing to pathologies such as multi-organ dysfunction and acute lung injury thus exerting a considerable impact on overall morbidity and mortality. In this study, we examine if transfusion is an independent predictor of mortality, or if outcomes are merely a result of the initial severity as predicted by Pediatric Risk of Mortality (PRISM) III, Pediatric Index of Mortality (PIM2), and day 1 Pediatric Logistic Organ Dysfunction (PELOD) scores.

**Methods:**

A single center retrospective study was conducted using data from a prospectively maintained transfusion database and center-specific data at our pediatric ICU between January 2009 and December 2012. Multivariate regression was used to control for the effects of clinical findings, therapy, and severity scores, with mortality as the dependent variable. Likelihood ratios and area under the curve were used to test the fidelity of severity scores by comparing transfused vs. non-transfused patients.

**Results:**

There were 4975 admissions that met entry criteria. In multivariate analysis, PRISM III scores and serum hemoglobin were significant predictors of transfusion (*p* < 0.05). Transfused and non-transfused subjects were distinctly disparate, so multivariate regression was used to control for differences. Severity scores, age, volume transfused, and vasoactive agents were significantly associated with mortality whereas hemoglobin was not. A substantial number of transfusions (45 %) occurred in the first 24 h, and patients transfused later (24–48 h) were more likely to die compared to this earlier time point. Likelihood ratio testing revealed statistically significant differences in severity scoring systems to predict mortality in transfused vs. non-transfused patients.

**Conclusions:**

This study suggests that RBC transfusion is an important risk factor that is statistically independent of severity. The timing of transfusions that related strongest to mortality remained outside the purview of severity scoring, as these happened beyond the timing of data collection for most scoring systems.

## Background

Transfusions are a necessary part of clinical practice, though they have very specific deleterious effects (e.g., anaphylaxis, transfusion related acute lung injury, transfusion associated circulatory overload) [1–3]. In addition, transfusions have poorly characterized but multi-systemic effects that are thrombogenic, immunosuppressive, and inflammatory in nature leading to deterioration independent of presenting illness [4–8]. Thus, red blood cell (RBC) transfusions could have direct and indirect effects by interacting with the presenting illness, leading to a significant impact on ICU morbidity and mortality [9–13].

The above pathophysiologic factors may explain why restrictive transfusion protocols have been shown to reduce transfusions without any negative impact on outcomes. Multiple prospective observational studies have examined the impact of RBC transfusions in critically ill patients [14, 15]. These studies have sought to establish the safety of lower hemoglobin thresholds rather than examine any specific negative impact or interactions of transfusion with critical illness. Lower hemoglobin thresholds have been established as safe by both adult and pediatric studies [14–16]. As thresholds drop, the patient’s clinical condition becomes more of a factor in making the decision to transfuse. Controlling for the confounding effects of the disease processes leading to transfusion in the ICU setting is difficult since healthy children require neither intensive care nor transfusion. We hypothesize that transfusion has effects that associate with mortality that are independent from severity of illness.

Therefore, we investigated whether transfusion status had any residual impact on length of stay (LOS) or mortality after controlling for well-established illness severity scores (Pediatric Risk of Mortality (PRISM) III, Pediatric Index of Mortality (PIM2), and Pediatric Logistic Organ Dysfunction (PELOD)), in addition to other variables. Severity scores provide us with predicted mortality as they were developed to enable comparative analysis of outcomes and benchmarking of care between different ICUs [17, 18]. These scores are derivatives of multiple physiological indices, laboratory values, and patient characteristics. If transfusion continues to have a significant association with outcomes after controlling for severity scores, this would suggest at a minimum that transfusion status captures aspects of acuity and risk not fully addressed by these scores.

## Methods

All pediatric patients (<18 years of age) admitted to our tertiary care pediatric intensive care unit (PICU) between January 2009 and December 2012 were included in the initial review. Data sources for the study included electronic medical records (CERNER; Kansas City, MO), Virtual PICU Systems, LLC database, and an Institutional Review Board (IRB)-approved prospective transfusion database. A wide range of clinical data are systematically captured in this database including diagnosis, transfusion trigger, pre-transfusion hemoglobin, timing of transfusion, volume and number of transfusions, and clinical course. IRB approval for this study was obtained prior to data collection. Further, none of the authors had any competing interests to declare.

There were two groups of patients for this study, the non-transfused group and the transfused group. The latter group included patients that received packed RBC transfusions only. Of note, all the blood used in our institution is leukocyte-reduced. Patients who received blood products other than RBCs were excluded to avoid confounding effects of non-RBC transfusions. If a patient received both RBCs and another product such as plasma, they were excluded as well. Patients were considered transfused if the transfusion occurred within 24 h prior to PICU admission, in order to capture all the intra-operative and emergency room transfusions, as well as during the PICU course. The time of the first transfusion was designated as the “time of transfusion,” but all RBC transfusions during the PICU course were considered for the total volume.

Post-operative cardiovascular admission patients, as well as patients that received extracorporeal support (e.g., ECMO, chronic renal replacement therapy), were excluded. In addition, the following patients were excluded due to low acuity: patients admitted to the PICU solely for the purposes of procedural sedation and patients admitted for relatively minor illnesses but requiring PICU status solely due to the need for continuation of home ventilator support.

Data collected included age at admission, gender, vasoactive agents used (y/n), reason for PICU admission (e.g., respiratory, trauma, gastrointestinal, cardiovascular, neurologic, oncologic), hemoglobin on admission <7 g/dL, transfused (y/n), indications for transfusion (e.g., active blood loss, hemodynamic instability, hypoxia, anemia, unspecified), time of transfusion (defined as <0 h (prior to PICU admission), 0 to <24 h, 24 to 48 h, >48 h), total volume transfused (all RBC transfusions during the PICU course), severity scores (PIM2, PRISM III, day 1 PELOD), PICU LOS (days), mechanical vent days, and mortality (30-day, overall).

Patients on chronic ventilator support were considered mechanically ventilated for the study if they needed higher ventilator settings than used at home and were classified as extubated when they got back to their baseline ventilator settings. Patients brought into the PICU intubated after a surgical procedure were not considered to be on mechanical ventilation unless the duration of support was >24 h.

Organ dysfunction was defined according to the criteria established by Wilkinson et al. [19] and subsequently modified by Proulx et al. [20]. Inotrope and vasopressor use were combined together as done by Gaies et al., who modified the original pressor scoring system by Ceneviva et al. [21]. For the purposes of this study, the use of both vasopressors and inotropes was classified together as vasoactive agents [22].

The PRISM III score is based on clinical and laboratory parameters assessed during the first 12 h and the PIM2 score on the first hour after admission to the PICU [23, 24]. The PELOD score, which considers 12 variables relating to six organ dysfunctions (cardiovascular, respiratory, hematological, neurological, hepatic, renal dysfunction), is recorded daily for the entire PICU admission or for the first 9 days of PICU stay, whichever comes first [25].

Data were analyzed using IBM SPSS Statistics v. 21.0 (Armonk, NY). Significance for all analyses was assessed at *p* < 0.05. Summary statistics was calculated for the data. Quantitative data are expressed as the mean ± SD, while nominal data are expressed as a percentage. Differences between the transfused and non-transfused groups for quantitative variables were determined using the *t* test, and nominal variables were compared using the *χ*^2^ test. The ANOVA procedure, with post hoc comparisons to investigate significant differences, was used to analyze differences among the different time points for time of first transfusion. These groups were defined as <0 h (prior to PICU admission), 0 to <24 h, 24 to 48 h, and >48 h.

Multiple regression analyses were performed to examine predictors of decision to transfuse, PICU LOS, and overall mortality. Dependent and independent variables for each regression model are shown in Table 1.

**Table 1:** Dependent and independent variables for each regression model

Dependent variable	Independent variables
Transfusion (y/n)	Hemoglobin concentration <7 g/dL, PIM2, PRISM III, day 1 PELOD, reason for PICU admission
PICU LOS (days)^a^	Age at admission, gender, transfusion, vasoactive agents, PIM2, PRISM III, day 1 PELOD, reason for PICU admission
Mortality (y/n)	Age at admission^c^, gender, volume transfused (mL/kg), hemoglobin concentration, PIM2 score, PRISM III score, vasoactive agents, PIM2, PRISM III, day 1 PELOD, reason for PICU admission
Mortality (y/n)^b^	Age at admission^c^, gender, time of transfusion, vasoactive agents, PIM2, PRISM III, day 1 PELOD, reason for PICU admission

*PICU* pediatric intensive care unit, *LOS* length of stay

^a^Due to non-normality of data, the natural log of PICU LOS was used

^b^Transfused patients only

^c^Ages were divided by 10 prior to analysis for ease of interpretation of the odds ratio

Two additional sets of analyses were performed to analyze the relationship between RBC transfusion and mortality. The first was to look at the difference in outcome measures between the transfused and non-transfused patients by receiver operating characteristic (ROC) curves using PRISM III, PIM2, and day 1 PELOD to predict mortality. The second analysis involved a comparison of likelihood ratios between the transfused and non-transfused groups. The cutoff points for the analyses were obtained using a series of logistic regression equations, with PRISM III scores, PIM2 scores, or day 1 PELOD scores as the independent variables, and mortality as the dependent variable. Comparisons between the transfused and non-transfused subjects for area under the curve (AUC) and likelihood ratios were performed.

## Results

### Demographic and clinical data

There were 5185 admissions to our PICU during the study period. Of these, 4975 met the inclusion criteria, with 536 (10.8 %) admissions resulting in transfusions for various indications. Demographic and clinical data are shown in Table 2. The overall mortality rate was 90/4975 (1.8 %). Overall mortality and mortality within 30 days of admission were both significantly higher in the transfused group. Non-transfused patients were younger than transfused patients, while the male to female ratio was very similar between the two groups. There were more patients with trauma, gastrointestinal, and cardiovascular pathology as a reason for PICU admission among the transfused patients compared to non-transfused. The PRISM III, PIM2, and day 1 PELOD scores were significantly higher in the transfused group. Variables indicating the level of support (use of vasoactive agents, ventilator days, and PICU LOS) were all significantly higher in the transfused group.

**Table 2:** Demographic and clinical data of the study sample

Variables	Non-transfused^#^	Transfused^^^	*p* value
Age at admission (months)	83.2 ± 74.6	92.4 ± 81.2	0.01
Sex (M/F)	2385/2054	289/247	0.98
Hemoglobin (g/dL)	12.0 ± 2.2	10.4 ± 2.4	<0.001
Reason for PICU admission			
Respiratory	1687/4439 (38 %)	95/536 (17.7 %)	<0.001
Cardiovascular	160/4439 (3.6 %)	70/536 (13.1 %)	<0.001
Gastrointestinal	134/4439 (3.0 %)	35/536 (6.5 %)	<0.001
Neurologic	703/4439 (15.8 %)	37/536 (6.9 %)	<0.001
Oncologic	160/4439 (3.6 %)	28/536 (5.2 %)	<0.06
Trauma	429/4439 (9.7 %)	106/536 (19.8 %)	<0.001
PRISM III score	2.5 ± 4.1	7.7 ± 9.0	<0.001
PIM2 score	−5.1 ± 1.5	−4.1 ± 1.8	<0.001
Day 1 PELOD	5.3 ± 5.2	9.7 ± 9.2	<0.001
Vasoactive agents	408/4439 (9.2 %)	94/536 (17.5 %)	<0.001
PICU LOS (days)	3.1 ± 5.9	9.5 ± 14.7	<0.001
Mechanical ventilation (days)	3.9 ± 8.9	9.1 ± 14.6	<0.001
Mortality *n* (%)	42/4439 (0.9 %)	48/536 (9.0 %)	<0.001
30-day mortality *n* (%)	40/4439 (0.9 %)	44/536 (8.2 %)	<0.001

*PICU* pediatric intensive care unit, *LOS* length of stay

^#^ *n* = 4439 for age at admission, PIM2, day 1 PELOD, PICU LOS; *n* = 1697 for hemoglobin level on admission; *n* = 4437 for PRISM III; *n* = 1131 for mechanical ventilation

^^^ *n* = 530 for age at admission and PIM2; *n* = 440 1697 for hemoglobin level on admission; *n* = 529 for PRISMIII; *n* = 536 for day 1 PELOD; *n* = 406 for PICU LOS; *n* = 304 for for mechanical ventilation

### Timing of first transfusion

Comparisons between the time points of first transfusion groups are shown in Table 3. The majority of transfusions (45 %) occurred within 0–<24 h following PICU admission, while 13 % occurred prior to PICU admission. The patients transfused prior to PICU admission were significantly older than those transfused at the other time points. The PRISM III and PIM2 scores were significantly lower in patients transfused prior to PICU admission compared to the other time intervals. All other time periods had comparable severity scores. Among the patients being transfused prior to PICU admission, 90 % were admitted for post-operative or trauma reasons. Overall and 30-day mortality were lowest for patients being transfused prior to PICU admission. Patients transfused at the 24–48 h time interval had the highest overall and 30-day mortality of the four groups.

**Table 3:** Patient characteristics by timing of transfusion relative to PICU admission

Timing of first transfusion	<0 h^^^	0 to <24 h^@^	24–48 h^$^	>48 h&	*p* value
Age at admission (months)	128 ± 74	91 ± 80	86 ± 87	80 ± 81	0.001*
Gender (females)	40 (56 %)	116 (48 %)	28 (37 %)	57 (40 %)	0.065
Indications for PRBC					
Active blood loss	5 (7 %)	81 (34 %)	29 (38 %)	32 (22 %)	<0.001
Hemodynamic instability	3 (4 %)	63 (26 %)	15 (20 %)	35 (24 %)	0.001
Hypoxia	0 (0 %)	9 (4 %)	3 (4 %)	11 (8 %)	0.053
Anemia	1 (1 %)	71 (30 %)	16 (21 %)	49 (34 %)	<0.001
Unspecified^a^	62 (87 %)	1 (0.4 %)	5 (7 %)	2 (1 %)	<0.001
Reason for PICU admission					
Cardiovascular	3 (4 %)	30 (13 %)	14 (18 %)	22 (15 %)	0.056
Post-operative	40 (56 %)	35 (15 %)	10 (13 %)	16 (11 %)	<0.001
Respiratory	1 (1 %)	39 (16 %)	18 (24 %)	37 (26 %)	<0.001
Trauma	24 (34 %)	44 (18 %)	16 (21 %)	21 (15 %)	0.009
Oncologic	0 (0 %)	21 (9 %)	1 (1 %)	5 (4 %)	0.003
Severity scores					
PRISM III score	3.7 ± 5.2	9.2 ± 10.3	8.0 ± 7.9	7.4 ± 8.1	<0.001*
PIM2 score	−5.1 ± 1.6	−4.0 ± 1.9	−3.8 ± 1.8	−4.0 ± 1.7	<0.001*
Day 1 PELOD	9.2 ± 7.2	14.2 ± 12	13.1 ± 11.9	12.6 ± 10.2	0.006*
Hemoglobin (g/dL)	9.1 ± 2.0	7.5 ± 2.1	8.2 ± 3.3	7.9 ± 1.4	<0.001**
Transfused amount (mL/kg)	12.4 ± 6.6	13.3 ± 7.3	13.1 ± 5.8	13.5 ± 5.5	0.679
Mortality	1/71 (1 %)	21/240 (9 %)	13/76 (17 %)	13/142 (9 %)	0.012
30-day mortality	1/71 (1 %)	21/240 (9 %)	12/76 (16 %)	10/142 (7 %)	0.016

*PRBC* packed red blood cells; *PICU* pediatric intensive care unit

^^^ *n* = 71 with the following exception: *n* = 49 for pre-transfusion Hb

^@^ *n* = 240 with the following exceptions: *n* = 239 for age at admission, PIM2, pre-transfusion Hb, and transfused amount; *n* = 238 for PRISM III

^$^ *n* = 76 with the following exceptions: *n* = 74 for PRISM III and PIM2

&*n* = 143 with the following exceptions: *n* = 140 for age at admission, PRISM III, and PIM2; *n* = 142 for day 1 PELOD and transfused amount; *n* = 139 for pre-transfusion hb

*<0 h group significantly different from all other groups, *p* < 0.01 (all other groups not significantly different from one another, *p* > 0.05)

**<0 h group significantly different from 0–<24 h group and >48 h group, *p* < 0.03

^a^Patients that had no reason identified for transfusion

### Decision to transfuse

The results of the regression procedure examining decision to transfuse are shown in Table 4. A serum hemoglobin <7 g/dL increased the chances of transfusion 50-fold. Independent of serum hemoglobin, for every 10-point increase in initial PRISM III scores, there was a 1.4-fold increase in the chance of RBC transfusion. Cardiovascular and trauma patients were more likely to be transfused than those with a respiratory diagnosis, while patients with a neurologic diagnosis were less likely to be transfused than patients with a respiratory diagnosis. PIM2 and day 1 PELOD scores were not associated with transfusion.

**Table 4:** Logistic regression analysis, with transfusion as the dependent variable (*n* = 2207)

Variable	Odds ratio	95 % confidence interval	*p* value
PRISM III score	1.04	1.01–1.07	0.006
Hemoglobin ≥7 g/dL	0.02	0.01–0.03	<0.001
Reason for PICU admission^a^			
Cardiovascular	2.26	1.39–3.66	0.001
Neurologic	0.58	0.35–0.96	0.034
Trauma	2.41	1.66–3.50	<0.001
Other^b^	1.32	0.96–1.83	0.093
Day 1 PELOD	1.02	0.997–1.04	0.106
PIM2 score	1.09	0.99–1.19	0.084

^a^Reason for PICU admission, odds ratio relative to respiratory (reference group)

^b^Other includes all other categories (e.g., endocrinologic, gastrointestinal, infectious, oncologic)

### PICU LOS

Several independent variables were predictive of longer PICU LOS (Table 5). Transfusion was associated with a 2.3-day increase in PICU LOS, while the use of vasoactive agents was associated with a 1.5-day increase in PICU LOS. Cardiovascular, neurologic, and trauma reasons for PICU admission were all associated with significantly shorter PICU LOS compared to patients admitted for respiratory reasons. Gender and age were not significant predictors of PICU LOS.

**Table 5:** Multiple regression analyses, with PICU LOS as the dependent variable (*n* = 4832)

Variables	β-coefficient	95 % confidence interval	*p* value
Dependent variable: ln(PICU LOS)^a^			
PIM2 score	0.138	0.118–0.159	<0.001
Transfusion	0.833	0.731–0.935	<0.001
Vasoactive agents	0.377	0.302–0.452	<0.001
Day 1 PELOD	0.013	0.008–0.018	<0.001
PRISM III score	−0.011	−0.018 to −0.003	0.017
Reason for PICU admission^b^			
Cardiovascular	−0.308	−0.447 to −0.170	<0.001
Neurologic	−0.239	−0.322 to −0.156	<0.001
Trauma	−0.441	−0.538 to −0.343	<0.001
Other^c^	−0.262	−0.330 to −0.195	<0.001
Gender	−0.027	−0.080 to −0.026	0.324
Age at admission (months)	0.000	0.000 to −0.000	0.716

^a^Due to the non-normality of the variable PICU LOS, the variable was transformed by taking its natural logarithm prior to analysis; the β-coefficients are interpreted by calculating the antilog, which for transfusion is e^0.833^, meaning that transfused subjects had a 2.3 day longer PICU LOS than non-transfused subjects

^b^Reason for PICU admission, odds ratio relative to respiratory (reference group)

^c^Other includes all other categories (e.g., endocrinologic, gastrointestinal, infectious, oncologic)

### Factors related to mortality

PRISM III and PIM2 scores and the use of vasoactive agents and RBC volume transfused were associated with mortality (Table 6). Serum hemoglobin levels were not significantly predictive in the model. Trauma patients were 4.4 times more likely to die, relative to the reference diagnosis, respiratory.

**Table 6:** Logistic regression analyses, with mortality as the dependent variable (*n* = 2208)

Variable	Odds ratio	95 % confidence interval	*p* value
PIM2 score	1.69	1.35–2.12	<0.001
PRISM III score	1.19	1.14–1.25	<0.001
Volume transfused (mL/kg)	1.08	1.03–1.13	0.001
Inotropes	2.99	1.40–6.41	0.005
Age at admission (months)^a^	0.96	0.92–1.01	0.144
Hemoglobin (g/dL)	1.07	0.93–1.22	0.366
Gender	1.64	0.81–3.31	0.170
Reason for PICU admission^b^			
Cardiovascular	2.45	0.80–7.53	0.117
Neurologic	0.96	0.28–3.32	0.948
Trauma	4.39	1.22–15.85	0.024
Other^c^	1.90	0.53–6.77	0.324

^a^All ages divided by 10 prior to analysis

^b^Reason for PICU admission, odds ratio relative to respiratory (reference group)

^c^Other includes all other categories (e.g., endocrinologic, gastrointestinal, infectious, oncologic)

### Timing of transfusion related to mortality

The results for the regression analysis examining mortality in the transfused group only are shown in Table 7. Although the overall effect of timing of transfusion was significant, only the 0- to <24-h group was different from the reference group (24–48 h), with a significantly decreased risk of mortality. Age at admission, vasoactive agents, PIM2, PRISM III, and day 1 PELOD scores were also significantly predictive of mortality. Cardiovascular patients were also associated with a significantly lower mortality, relative to the reference diagnosis, respiratory.

**Table 7:** Logistic regression analyses with only transfused subjects, with mortality as the dependent variable (*n* = 514)

Variable	Odds ratio	95 % confidence interval	*p* value
PIM2 score	1.59	1.10–2.28	0.013
PRISM III score	1.17	1.07–1.28	0.001
Age^a^	0.90	0.82–0.98	0.016
Transfusion^b^			
<0 h	0.06	0.002–2.00	0.118
0 h to <24 h	0.08	0.02–0.32	<0.001
>48 h	0.36	0.10–1.33	0.124
Gender	2.80	0.95–8.25	0.062
Vasoactive agents	4.94	1.37–17.86	0.015
Day 1 PELOD score	1.06	0.99–1.13	0.117
Reason for PICU admission^c^			
Cardiovascular	0.14	0.03–0.66	0.013
Neurologic	2.37	0.41–13.78	0.338
Trauma	0.81	0.12–5.42	0.830
Other^d^	1.27	0.27–5.90	0.759

^a^All ages divided by 10 prior to analysis

^b^Refers to timing of transfusion relative to ICU admission; <0 h group was transfused prior to ICU admission; the reference group was transfused within the 24–48 h time frame

^c^Reason for PICU admission, odds ratio relative to respiratory (reference group)

^d^Other includes all other categories (e.g., endocrinologic, gastrointestinal, infectious, oncologic)

### AUC and likelihood ratios

Transfusion did not significantly modify the discriminatory performance (ability to predict mortality) of the three severity scores (Table 7). However, the positive likelihood ratios (LR+) calculated for all three scores for the non-transfused patients were all significantly higher than the LR+ for the transfused patients. The differences ranged from sixfold higher for day 1 PELOD scores to over tenfold higher for PIM2 scores. There were no significant differences found between transfused and non-transfused patients for negative likelihood ratios (LR−) for any of the three scores.

## Discussion

The goal of this study was to test the hypothesis that transfusion is an independent predictor of mortality and that this relationship is independent of severity of illness. Our data showed that increases in transfusion volume, PRISM III score, and PIM2 score, as well as use of vasoactive agents, were significantly predictive of mortality. Additionally, the LR+ calculated for all three scores indicated a significant difference between the transfused and the non-transfused patients. Based upon these data, one could hypothesize that the predictive ability of all three severity scores could be improved by accounting for transfusion status.

As expected, low serum hemoglobin (<7 g/dL) markedly and significantly increased the odds of a transfusion. Despite this strong relationship, hemoglobin concentration was not a significant predictor of mortality. It is often the sicker patient that needs a transfusion, which is supported by our findings that a higher PRISM III score was independently associated with transfusion. But this creates a dilemma as univariate analyses demonstrated numerous differences between the transfused and non-transfused cohort. Regression modeling was used to control for those differences so that we could examine transfusion as an independent risk factor for mortality.

Both AUC and likelihood ratios were used to examine the potential for RBC transfusions to modify the predictive ability of severity scoring systems (Table 8). Whereas AUCs are derived from a large number of decision points [26], likelihood ratios use an optimally derived cut-point to express the change in the risk that a disease process is present at a given measurement and has been previously used to examine the predictive capability of severity scores [27–30]. The likelihood ratio for both transfused and not transfused patients should be similar if transfusion is merely an indication of severity. What we found instead was, although severity scores predict mortality rather well (LR+ values >10) for both groups, there was a statistically significant difference in the positive likelihood ratios. This suggests that transfusions introduce a degree of unpredictability by exhibiting significantly lower LR+. The LR+ tells us how much to increase the probability of mortality if the test is positive. The inference is severity scores predict mortality better in the non-transfused group. The significant difference in LR+ and the strength of transfusion as an independent risk factor in regression modeling lead us to believe that transfusion adds risk independent of disease severity.

**Table 8:** AUC and likelihood ratios in the prediction of mortality

Severity Score	Non-transfused	Transfused	*p* value
	AUC	AUC	
PRISM III	0.91 (0.84–0.97)	0.89 (0.83–0.95)	0.74
PIM2	0.93 (0.89–0.97)	0.88 (0.82–0.94)	0.19
Day 1 PELOD	0.92 (0.86–0.98)	0.90 (0.84–0.95)	0.61
	LR+ (95 % CI)	LR+ (95 % CI)	
PRISM III	471 (166–1332)	64 (23–176)	0.007
PIM2	471 (167–1333)	38 (15–97)	0.0004
Day 1 PELOD	314 (120–825)	53 (18–143)	0.01
	LR− (95 % CI)	LR− (95 % CI)	
PRISM III	0.57 (0.44–0.74)	0.39 (0.25–0.59)	0.12
PIM2	0.57 (0.44–0.74)	0.55 (0.41–0.74)	0.84
Day 1 PELOD	0.64 (0.51–0.81)	0.52 (0.38–0.72)	0.28

*AUC* area under the curve from a receiver operating characteristic (ROC) analysis; *LR+* positive likelihood ratio, *LR−* negative likelihood ratio; *CI* confidence interval

If RBC transfusions by themselves are independent predictors of mortality as suggested by our study, one could propose that existing severity scores should be modified to account for transfusion status. With this in mind, we examined if any relationship existed between the timing of transfusions and mortality. This timing is critical, as the relationship of transfusion with mortality would have to be shown to occur early enough in the course of illness to be available for inclusion into the scoring of disease severity. Our data indicate, however, that the groups with most mortality are those that have transfusions later in their course (24–48 h). One possible explanation for the worse mortality in patients who receive their first transfusion later could be that, at least in adults, it has been shown that organ failure which occurs early in the ICU course and therapies early in that first 48 h could influence the patient’s recovery [31]. One could speculate that this is likely a vulnerable period during which time patients are precariously poised to either improve or deteriorate. One could hypothesize that patients transfused early in their PICU course do better because there is less likelihood for an established inflammatory process and the capacity for transfusion to have a negative impact on mortality is diminished.

### Limitations

Both the Virtual PICU Systems database and our transfusion database contain prospectively maintained data, but the study hypothesis was conceived only after the information was collected. The single center retrospective design limits our ability to account for the way transfusion was applied to critically ill patients. Another limitation is that, severity scores such as PIM2 and PRISM III were not designed to examine therapeutic interventions that most often occur after the first day of ICU admission. Thus, at best, severity scores remain surrogates for acuity and are incomplete depictions of true severity. We excluded certain high mortality groups such as patients receiving extracorporeal support with ECMO and chronic renal replacement therapy because these patients often received other blood products. In addition, the decision to transfuse in these patients is not one that is clinically based but rather protocol driven.

## Conclusions

We showed RBC transfusion to be an important risk factor that is statistically independent of severity in critically ill patients. The transfusions that seem to associate most with mortality happen after that first 24–48 h. Currently, severity scores do not account for RBC transfusions but the predictive ability of severity scores could perhaps be improved by accounting for them. However, this remains outside of the timing of data collection for most severity scores except for PELOD scores.

### Availability of supporting data

There are strict institutional rules controlling our ability to create an external repository of data, even if anonymized. If there are questions about the validity or integrity of our data, the corresponding author will address them as requested.

## Abbreviations

RBC: red blood cell
PIM2: Pediatric Index of Mortality
PRISM: Pediatric Risk of Mortality
PELOD: Pediatric Logistic Organ Dysfunction Score
PICU: Pediatric Intensive Care Unit
ECMO: extracorporeal membrane oxygenation
IRB: Institutional Review Board (serves as board for ethical conduct of research)
VPS: Virtual PICU Systems
